# Glenohumeral joint motion after subscapularis tendon repair: an analysis of cadaver shoulder models

**DOI:** 10.1186/1749-799X-9-41

**Published:** 2014-05-23

**Authors:** Teiichi Sano, Mitsuhiro Aoki, Yoshitaka Tanaka, Tomoki Izumi, Mineko Fujimiya, Toshihiko Yamashita

**Affiliations:** 1 Department of Orthopaedic Surgery, Shizuoka General Hospital, 4-27-1 Kitaandou, Aoi-ku, Shizuoka-city, Shizuoka 420-8527, Japan; 2 Department of Orthopaedic Surgery, Health Sciences University of Hokkaido School of Rehabilitation Sciences, 1757 Kanazawa, Tobetsu-cho, Ishikari-gun, Hokkaido 061-0293, Japan; 3 Orthopaedic Trauma and Microsurgery Center, Seikeikai Hospital, 4-2-10, Kouryounaka-machi, Sakai-ku, Sakai-city, Osaka 590-0024, Japan; 4 Doctoral Course of Physical Therapy, Graduate School of Health Sciences, Sapporo Medical University, S1 W17, Chuo-ku, Sapporo-city, Hokkaido 060-8556, Japan; 5 Department of Anatomy Section 2, Sapporo Medical University School of Medicine, S1 W17, Chuo-ku, Sapporo-city, Hokkaido 060-8556, Japan; 6 Department of Orthopaedic Surgery, Sapporo Medical University School of Medicine, S1 W17, Chuo-ku, Sapporo-city, Hokkaido 060-8556, Japan

**Keywords:** Subscapularis tendon repair, Range of motion, Cadaver study

## Abstract

**Background:**

As for the surgical treatment of the rotator cuff tears, the subscapularis tendon tears have recently received much attention for the mini-open or arthroscopic repair. The results of surgical repair for the subscapularis tendon tear are satisfactory, but the range of external rotation is reported to be restricted after the repair. The purpose of this study was to evaluate the range of glenohumeral joint motion after repairs of various sizes of subscapularis tendon tears.

**Methods:**

Using eight fresh frozen human cadaveric shoulders (mean age at death, 81.5 years), three sizes of subscapularis tendon tear (small, medium, and large) were made and then repaired. With the scapula fixed to the wooden jig, the end-range of glenohumeral motion was measured with passive movement applied through 1.0-Nm torque in the directions of scapular elevation, flexion, abduction, extension, horizontal abduction, and horizontal adduction. The passive end-ranges of external and internal rotation in various positions with rotational torque of 1.0 Nm were also measured. Differences in the ranges among the three type tears were analyzed.

**Results:**

As tear size increased, range of glenohumeral motion in horizontal abduction after repair decreased gradually and was significantly decreased with the large size tear (*P* < 0.01). The end-range of external rotation decreased progressively with increasing tear size in every glenohumeral position. The prominent decrease in external rotation (around 40° reduction from intact shoulders) was observed in shoulders after repair of large size tear at 30° to 60° of scapular elevation and abduction.

**Conclusions:**

As the size of the subscapularis tendon tear increased, the passive ranges of horizontal abduction and external rotation of the glenohumeral joint after repair decreased significantly. In shoulders with a subscapularis tendon tear, it is necessary to consider the reduction of external rotation depending on tear size.

## Background

As for the surgical treatment of the rotator cuff tears, the subscapularis tendon tears have recently received much attention for the mini-open or arthroscopic repair [1-5]. The results of surgical repair for the subscapularis tendon tear are satisfactory, but the range of external rotation is reported to be restricted after the repair [1,3,6]. In a study using cadaveric shoulders, Muraki et al. reported that when tears of the supraspinatus tendon are repaired, passive shoulder range of motion was restricted significantly [7]. It is also thought that the repair of subscapularis tendon tear affect the shoulder motion [1,3,6], but no studies have investigated the reduction in glenohumeral joint motion. Lafosse et al. reported on a series of 17 patients who were managed with arthroscopic repair of an isolated subscapularis tear [8]. They indicated that postoperative range of motion on external rotation was decreased; however, postoperative Constant score and University of California at Los Angeles score were significantly improved. Adams et al. also reported on a series of 40 patients with arthroscopic subscapularis repairs who were followed for an average of 5 years [1]. They also indicated that postoperative range of motion on external rotation was significantly decreased; however, postoperative visual analog scale scores, modified University of California at Los Angeles score, and American Shoulder and Elbow Society scores were significantly improved. Thus, it is reported that the clinical outcome was improved even if the range of external rotation decreased after the subscapularis tendon repair. For this reason, it is thought that pain and muscle strength are improved after tendon repair. However, we lack the specific data that show how the external rotation movability of the glenohumeral joint changes after a tendon repair. Gerber et al. reported on a series of 16 patients with isolated subscapularis repairs who were followed for a minimum of 2 years [9]. They reported that 18.7% of patients had required arthroscopic release because of deficit of external rotation of at least 30°. The purpose of this study was to evaluate the range of glenohumeral joint motion after repairs of three sizes of subscapularis tendon tears with suture anchors using fresh frozen cadaveric shoulders. We hypothesized that the range of glenohumeral motion after subscapularis tendon repair decreased as tear size increased.

## Materials and methods

### Preparation of specimens

Eight fresh frozen human cadaveric shoulders with no previous shoulder surgery on history were used in this study. The shoulders were obtained from the right side of male specimens with a mean age of 81.5 years at death (range, 71 to 93 years). The frozen shoulders were thawed to room temperature (22°C) overnight prior to experiment. Each specimen was disarticulated at the scapulothoracic articulation, the clavicle was removed at the acromioclavicular joint, and the humerus was cut at the mid-diaphyseal part distal to the deltoid muscle attachment after titanium wire marking for the forearm direction. The skin and subcutaneous tissue were excised, leaving the rotator cuff muscles and tendons, the glenohumeral capsule, the long head of the biceps, and the coracoacromial and coracohumeral ligaments intact. Specimens with rotator cuff tears, ligament damage, and glenohumeral osteoarthritis were excluded.

### Testing apparatus

The scapula was fixed to the custom-designed wooden jig with free passive motion of the glenohumeral joint. The anterior surface of the scapula of the specimen was placed on the frontal face of the wooden jig, and the medial border of the scapula was placed perpendicular to the ground. The coracoid process and inferior and superior angles of the scapula were fixed with titanium screws. The #2 thread (Ethibond, Ethicon Inc, Somerville, NJ, USA) was passed through medial edge of each rotator cuff tendon in a modified Kessler fashion and routed through low-friction pulleys so that the thread was in the line of action for each of the muscles. To center the humeral head in the glenoid fossa during the test, a 10-N load was applied to the subscapularis tendon, a 5-N load was applied to the supraspinatus tendon, and 10-N loads were applied to the infraspinatus and teres minor tendons through the #2 Ethibond thread attached to each tendon. These loads were chosen based on previous cadaveric studies [10,11]. No load was applied to the long head of the biceps. An intramedullary acrylic rod was inserted into the humeral shaft and fixed with two titanium pins. Aligned with titanium wire marking for shoulder rotation, a carbon rod was inserted to the middle of the humerus, perpendicular to the humeral shaft parallel with the direction of the forearm. The specimen was kept moist through the experiment, which was performed at room temperature (22°C), with a spray of saline solution applied every 10 to 20 min.

**Figure 1:**
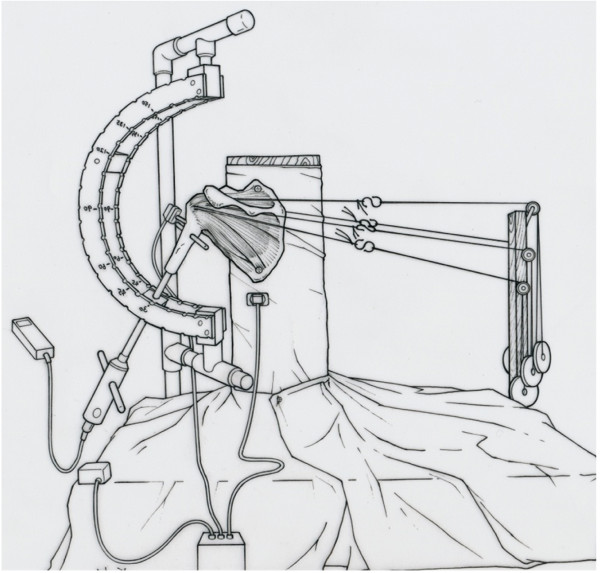
**Schematic illustration shows the experimental setup.** A scapula is fixed to the custom-designed wooden jig. A 6-degrees-of-freedom 3SPACE FASTRAK electromagnetic tracking device is used to measure the range of motion of the glenohumeral joint. The torque gauge is attached to the distal end of the intramedullary acrylic rod to add rotational torque to the humeral head. A carbon rod has been inserted to the middle of the humerus, perpendicular to the humeral shaft, to be used as a reference to indicate the rotational angle.

The wooden jig comprised a wooden board, a square post/column, and double semicircular frames with two columns. The distal tip of the intramedullary acrylic rod slid in hemicircular arms of the jig and placed the humerus in a given plane (such as the scapular plane) and angle (0°, 30°, 60°, and 90°) of elevation. This device also allowed the humerus to be placed at a given angle of internal and external rotation of the glenohumeral joint (Figure 1).

### Measurement device

A 6-degrees-of-freedom 3SPACE FASTRAK electromagnetic tracking device (POLHEMUS, Colchester, VT, USA) was used to measure the range of motion of the glenohumeral joint [7,11]. This device enabled measurement of the three-dimensional position and orientation of the receivers relative to the transmitter that generated an electromagnetic field in the experimental space. The transmitter was mounted in a fixed position to a nonmetallic stand, and two receivers were placed on the wooden jig and middle portion of the humerus. The three-dimensional space was defined as x, y, and z vectors, such that the *z*-axis was perpendicular to the ground, the *y*-axis was perpendicular to the *z*-axis in the scapular plane, and the *x*-axis was perpendicular to the *z*- and *y*-axes in the horizontal plane.

The torque gauge (RX-T portable torque gauge, AIKOH Engineering, Osaka, Japan) was attached to the distal end of the intramedullary acrylic rod to add rotational torque to the humeral shaft. The measurement range, minimal value, and accuracy of this torque gauge were 10 Nm, 0.001 Nm, and ±1%, respectively, as reported by the manufacturer.

### Experimental conditions

**Figure 2:**
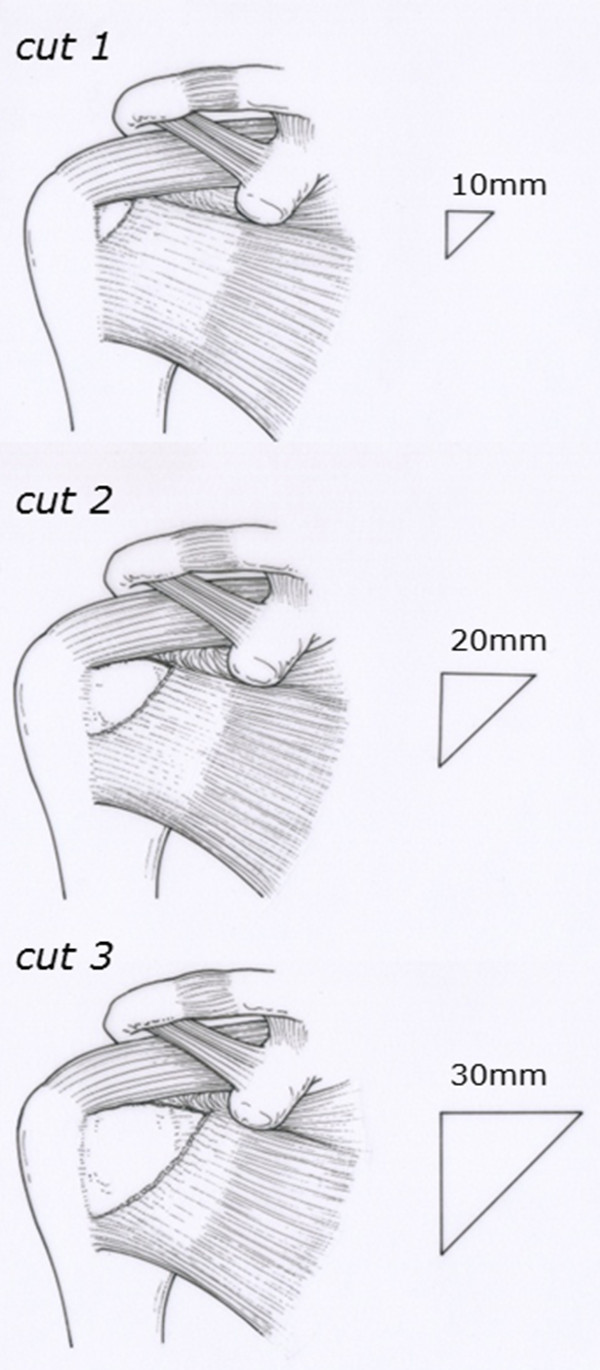
**Three graded, triangular full thickness tendon excisions.** Of 10 × 10, 20 × 20, and 30 × 30 mm were made in the superior-lateral aspect of the subscapularis tendon at the attachment of the lesser tuberosity and repaired. These were defined as cut 1, cut 2, and cut 3 models, respectively.

**Figure 3:**
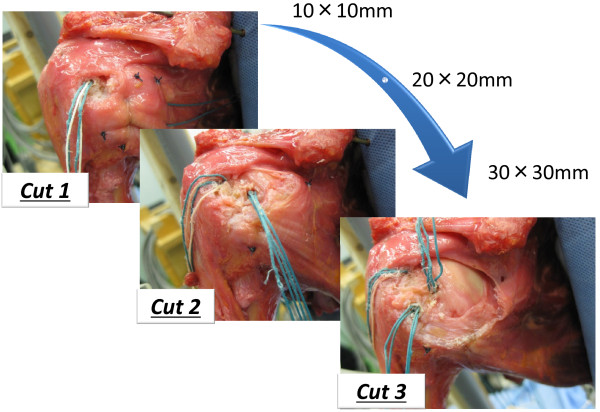
**Position of metallic suture anchors.** Metallic suture anchors loaded with #2 Ethibond threads were used to repair the torn subscapularis tendon. In all models, the torn edge was reinforced with #2-0 Ethibond threads around the circumference.

In this experiment, three sizes of full thickness subscapularis tendon tears were made (small size for cut 1, middle size for cut 2, and large size for cut 3). Based on the previous anatomic [12,13] and clinical [3,4] studies, three graded, triangular full thickness excisions of 10 × 10, 20 × 20, and 30 × 30 mm tendon were progressively made in the superior-lateral aspect of the subscapularis tendon at the attachment of the lesser tuberosity (Figure 2) and repaired. When we put threads through the subscapularis tendon, we simulated a repair method in the operation as much as possible. We pulled out a stump of the subscapularis tendon to most superior-lateral position and covered the footprint of the lesser tuberosity with lowest tension. We passed the suture through the 10 mm inside from a stump of the subscapularis tendon. To simulate arthroscopic tendon repair [14], in the cut 1 model, a first metallic suture anchor (Fastin RC suture anchor, Mitek, MA, USA) loaded with two pairs of #2 Ethibond threads was placed in the most superior-lateral position of the lesser tuberosity, just medial of the intertubercular groove, and the threads were passed through so as to penetrate the torn subscapularis tendon with mattress (Masson-Allen) sutures. In the cut 2 model, an additional metallic suture anchor was placed just lateral of the articular surface of the humeral head, with an interval between the two anchors of about 10 mm, and the threads were passed through the torn subscapularis tendon with sutures. In the cut 3 model, an additional metallic suture anchor was placed in the lateral margin of the lesser tuberosity, 20 mm from the first metallic suture anchor, and the sutures were passed through the torn subscapularis tendon with sutures. In all models, the torn edge was reinforced with #2-0 Ethibond threads with circumference, so that the torn subscapularis tendons were repaired to the original subscapularis footprint of the lesser tuberosity. Using the specimens, we simulated intact shoulder, cut1, cut2, and cut3 type full thickness tendon tear models, and then after repair, the range of glenohumeral motion was measured accordingly (Figure 3).

### Setting of the anatomical axes

The line of the intramedullary acrylic rod that was inserted into the humeral shaft was set as the longitudinal line of the humerus, and the line of the carbon rod that was inserted perpendicular to the humeral shaft was set as the rotational line of the humerus. Neutral rotation was defined as 30° of external rotation relative to the sagittal plane of the scapula that demonstrated neutral rotation relative to the trunk [11].

Glenohumeral joint motions were set on the basis of the fixed scapula. Scapular elevation, flexion, abduction, and extension were set as elevation in the scapular plane, elevation 60° anteriorly from the scapular plane, elevation 30° posteriorly from the scapular plane, and elevation 90° posteriorly from the scapular plane. The reference position was 0° of scapular elevation and 30° of external rotation relative to the sagittal plane of the scapula, which was demonstrated neutral rotation relative to the trunk [11]. Horizontal abduction and horizontal adduction were set as motion in the horizontal plane at the 60° of abduction, and the reference position was with the glenohumeral joint at 60° of abduction and neutral rotation.

### Motion measurements

In this experiment, the range of (1) glenohumeral joint motion and (2) external and internal rotation with the glenohumeral joint at the various predetermined positions after repair were measured. Passive movement was applied through 1.0-Nm torque in the directions of scapular elevation, flexion, abduction, extension, horizontal abduction, and horizontal adduction. The ranges of external and internal rotation at 0°, 30°, 60°, and 90° of scapular elevation, flexion, and abduction and at 0° and 30° of extension with rotational torque of 1.0 Nm were also measured. In the previous cadaveric studies [11,15] and our pilot study, the several values of the applied torque was used. Therefore, based on results of our pilot study and previous clinical study [15], the torque was determined to be 1.0 Nm. In the preliminary experiments, there was high repeatability of the range of motion at the glenohumeral joint with an applied torque of 1.0 Nm. In this study, no loosening of the repair was observed under 1.0 Nm joint torque.

Because the scapula was disarticulated from the thorax and fixed to the jig, 0°, 30°, 60°, and 90° of elevation relative to the scapula corresponded to 0°, 45°, 90°, and 135° of elevation relative to the trunk *in vivo*, respectively [16,17]. The measurements were performed three times in each position.

As for this study, the approval of the ethical review board of the university was obtained.

### Statistical analysis

One-way repeated measures analysis of variance was used to determine differences in the range of motion among the intact, cut 1, cut 2, and cut 3 tear models. The Bonferroni multiple comparisons test was used as a *post hoc* test to account for multiple comparisons. Student *t* test was used to compare the range of motion at every glenohumeral joint angle. The level of significance was set at *P* < 0.05. The statistical analyses were carried out with SPSS for Windows version 11.5 J (SPSS Japan Inc., Tokyo, Japan).

## Results

**Figure 4:**
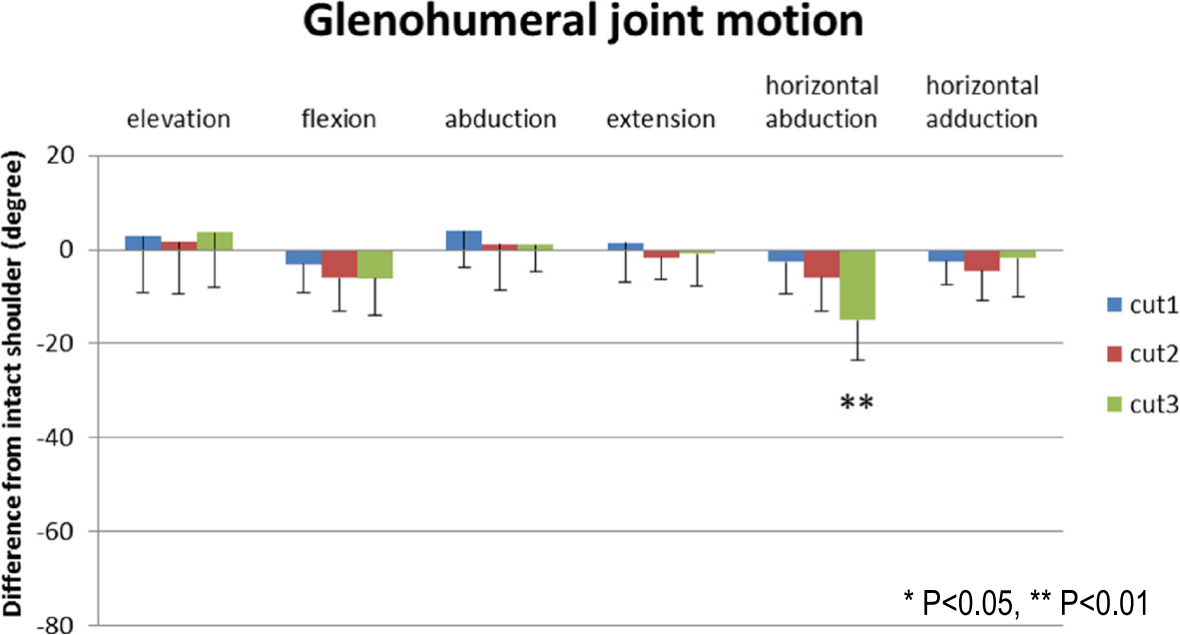
**The decrease of passive ranges of glenohumeral motion.** The range of horizontal abduction is significantly smaller with increasing tear size. *P < 0.05, **P < 0.01.

The changes of glenohumeral joint motion after each repair relative to intact specimens are presented in Figure 4. The passive ranges of scapular elevation, flexion, abduction, extension, and horizontal adduction after the repair of the subscapularis tendon were not significantly influenced by tear size. As the tear size increased, the range of horizontal abduction after the repair decreased and was significantly decreased in the cut 3 model compared with the intact shoulders (*P* < 0.01).

**Figure 5:**
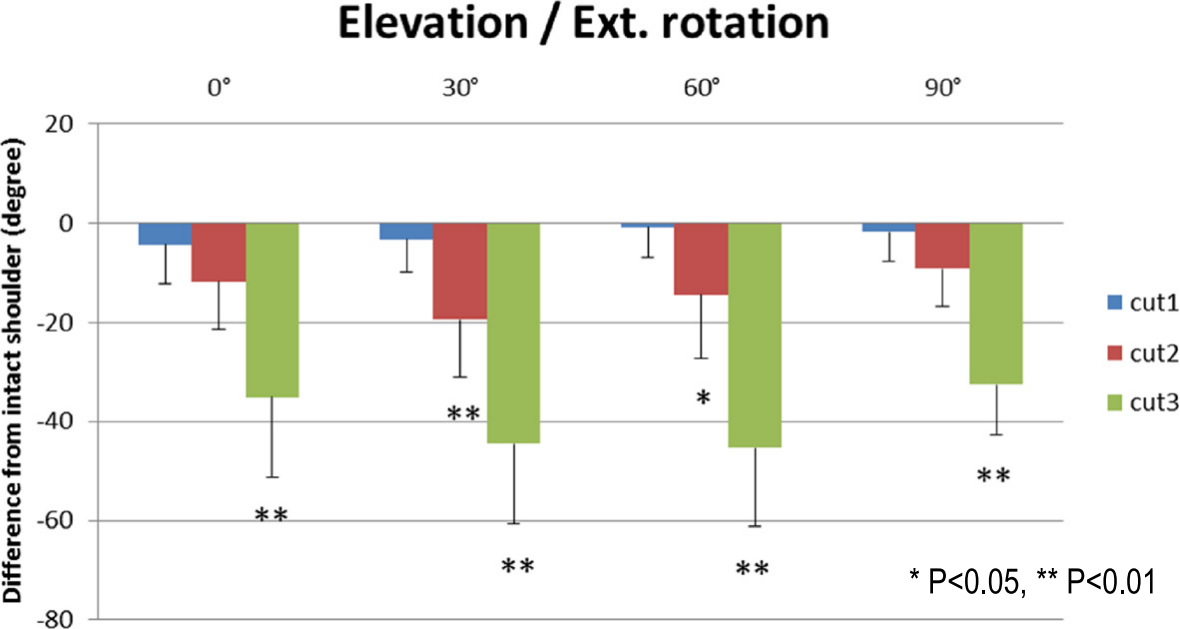
**The decrease of passive ranges of external rotation at scapular elevation.** The range of external rotation is progressively and significantly smaller with increasing tear size in every determined position of the arm. *P < 0.05, **P < 0.01.

**Figure 6:**
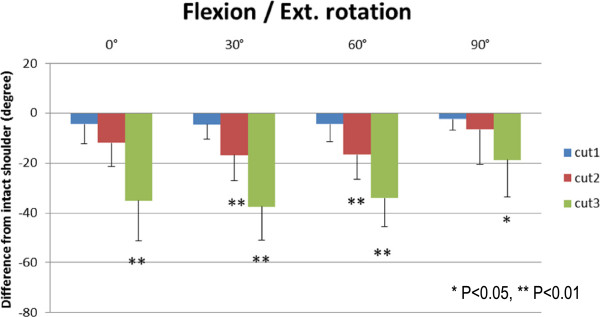
The decrease of passive ranges of external rotation at flexion.

**Figure 7:**
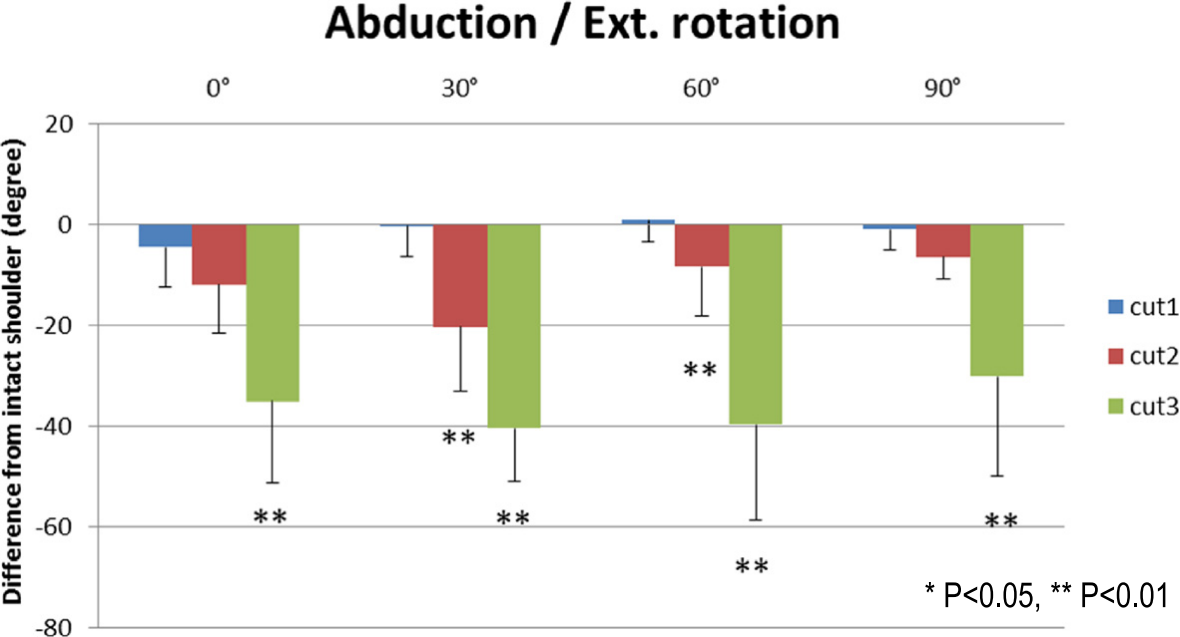
The decrease of passive ranges of external rotation at abduction.

**Figure 8:**
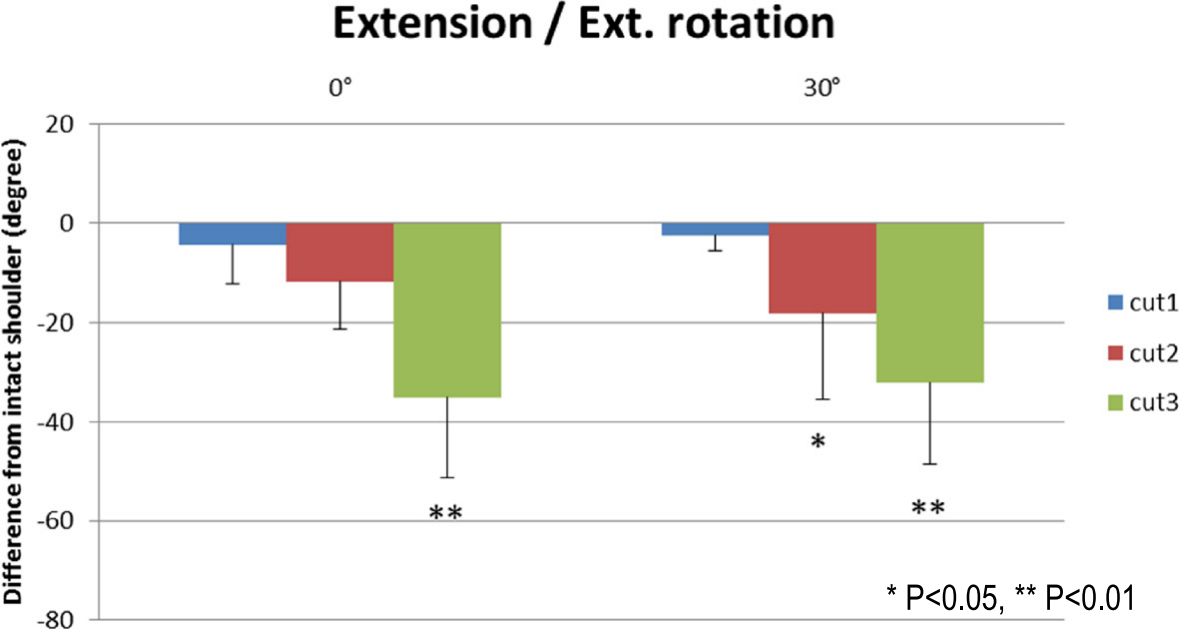
The decrease of passive ranges of external rotation at extension.

Results of the range of external rotation are presented in Figures 5, 6, 7, and 8. The range of external rotation became progressively smaller with increasing tear size in every predetermined position of the glenohumeral joint, especially in cut 3 model, they were significantly less than control intact shoulders.

No significant difference in internal rotation due to tear size was found in any of the glenohumeral joint positions.

## Discussion

Surgery for rotator cuff tears has been performed not only for postero-superior rotator cuff tears that include the supraspinatus and/or infraspinatus tendon but also for subscapularis tendon tears. In previous studies, the incidence rate of subscapularis tendon tear was 5.4% to 49.4% [2,6,8], and hyper-extension and hyper-external rotation forces by a direct force, sports injuries, and traffic accidents were reported as the injury mechanisms [1,9,18]. Gerber et al. reported on a series of sixteen patients with isolated subscapularis repairs who were followed for a minimum of two years. They reported that 18.7% of patients had required arthroscopic release because of deficit of external rotation of at least 30° [9]. It has been reported that the range of motion of external rotation is restricted after the repair of the subscapularis tendon tear [3,8], but no previous studies have investigated in detail how the difference in the size of full thickness subscapularis tendon tear affects glenohumeral joint motion after the repair.

In this study, a subscapularis tendon tear model of three different sizes was simulated, and passive range of motion on the glenohumeral joint motion after the repair was evaluated. The difference with previous researches and this study comes from the following two points. First, we made three sizes of subscapularis tendon tear based on clinical findings and previous anatomical and clinical studies. Second, we simulated all the basic motion of glenohumeral joint such as elevation, flexion, abduction, extension, horizontal abduction, horizontal adduction, and external and internal rotation of various positions. There were no previous studies which examined all these motions. As the size of the subscapularis tendon tear increased, the passive ranges of horizontal abduction and external rotation in multiple positions after the repair were decreased, and this tendency was marked in the cut 3 model. In the previous studies, repair of the shortened rotator cuff tendon [7,19], increased loading to the tendon [15], and contracture of the capsule [20] were reported as the causes of restriction of glenohumeral joint motion after rotator cuff tendon repair. Muraki et al. reported in a cadaveric study that repair of the shortened rotator cuff tendon could reduce the glenohumeral range of motion because of tightness of the tendon [7]. In the present study as well, it appears that the range of horizontal abduction and external rotation in all positions after repair was limited because surgical repair of the retracted subscapularis tendon increased the tension on the tendon. Because the lesser tubercle of the humerus migrates backward in the horizontal abduction, subscapularis tendon is towed. Thus, the range of motion decreases after subscapularis tendon repair with shortening of the tendon. Whereas, there is little migration of the lesser tuberosity of humerus in the elevation, abduction, and extension, and it is inferred that the range of motion does not change because subscapularis tendon is not towed. Kuhn et al. reported that increased loading to the subscapularis tendon was a factor in the limitation of the external rotation of the shoulder joint because of increasing of the rotational torque [15]. Flury et al. reported on a series of 73 patients with subscapularis repair [6]. They indicated that postoperative range of motion on external rotation was decreased; however, range of motion of elevation and abduction were improved after subscapularis repair. This report supports our results.

In shoulders with a repaired subscapularis tendon tear, ranges of motion of horizontal abduction and external rotation are decreased according to the size of the tendon tear. Therefore, it is necessary to consider the repair construct. Particularly in the case of suture of a large tendon defect, which is the scenario assumed for cut 3 model, caution must be exercised to avoid re-tears.

Harryman et al. reported that the size of the tear at the time of the repair correlated with the functional outcome [21]. Patients who have free shoulder range of motion except restriction of external rotation and horizontal abduction can bring food to the mouth with hands, write letters, type key board, comb hair, and perform perineal care [7]. The activity that they cannot conduct is considered to outstretch hands and throw objects. Therefore, results of our study are important to conduct physiotherapy in patients who required external rotation of the shoulder.

The following are considered the limitations of this study. First, this cadaveric study simulated the glenohumeral joint motion just after the repair of subscapularis tendon tear and did not replicate the healing environment of the living body. Physical therapy and stretching exercises could be used to increase the range of motion in the long postoperative term. After having started postoperative exercise therapy, we did not perform the measurement corresponding to the rotator cuff of which tensile strength increased over time. Second, the average age of the specimens was relatively high, and tendinous extensibility may have been decreased. Third, because the scapula was transected from the thorax, only the kinetics of glenohumeral joint motion was analyzed.

## Conclusions

As the size of the subscapularis tendon tear increased, the passive ranges of horizontal abduction and external rotation after repair were decreased. Results of our study are important to conduct physiotherapy in patients who required external rotation of the shoulder.

## Competing interests

The authors declare that they have no competing interests.

## Authors’ contributions

TS, M.D. is a researcher who performed experiment, collected and analyzed data, and wrote the manuscript. MA, M.D., Ph.D. is the corresponding author who designed and edited the manuscript. YT, M.D., Ph.D. and TI, P.T., Ph.D. are researchers who assisted this experiment. MF, M.D., Ph.D. is a contributor who provided cadaver specimens. TY, M.D., Ph.D. is a director who organized this experiment. All authors read and approved the final manuscript.
